# Long-lasting responses with chemotherapy followed by T-cell therapy in recurrent or metastatic EBV-related nasopharyngeal carcinoma

**DOI:** 10.3389/fimmu.2023.1208475

**Published:** 2023-07-11

**Authors:** Simona Secondino, Paolo Pedrazzoli, Sabrina Basso, Paolo Bossi, Alba Bianco, Ilaria Imarisio, Anna Pagani, Marica De Cicco, Stella Muscianisi, Michela Casanova, Carlo Morosi, Cristiana Bergamini, Marco Benazzo, Maria Cossu Rocca, Cesare Perotti, Fausto Baldanti, Marco Zecca, Lisa F. Licitra, Patrizia Comoli

**Affiliations:** ^1^ Department of Internal Medicine and Medical Therapy, University of Pavia, Pavia, Italy; ^2^ Department of Oncology, Fondazione Istituto di Ricerca e Cura a Carattere Scientifico (IRCCS) Policlinico S. Matteo, Pavia, Italy; ^3^ Cellular Therapy & Immunobiology Working Party, European Bone Marrow Transplantation (EBMT), Leiden, Netherlands; ^4^ Pediatric Hematology/Oncology, Fondazione Istituto di Ricerca e Cura a Carattere Scientifico (IRCCS) Policlinico S. Matteo, Pavia, Italy; ^5^ Cell Factory, Fondazione Istituto di Ricerca e Cura a Carattere Scientifico (IRCCS) Policlinico S. Matteo, Pavia, Italy; ^6^ Department of Medical Oncology, University of Brescia-Azienda Socio-Sanitaria Territoriale (ASST) Spedali Civili, Brescia, Italy; ^7^ Pediatric Oncology, Fondazione Istituto di Ricerca e Cura a Carattere Scientifico (IRCCS) Istituto Nazionale dei Tumori, Milan, Italy; ^8^ Radiology Unit, Fondazione Istituto di Ricerca e Cura a Carattere Scientifico (IRCCS) Istituto Nazionale dei Tumori, Milan, Italy; ^9^ Head and Neck Medical Oncology Unit, Fondazione Istituto di Ricerca e Cura a Carattere Scientifico (IRCCS) Istituto Nazionale dei Tumori, Milan, Italy; ^10^ Department of Clinical, Surgical, Diagnostic and Pediatric Sciences, University of Pavia, Pavia, Italy; ^11^ Otolaryngology - Head and Neck Surgery, Fondazione Istituto di Ricerca e Cura a Carattere Scientifico (IRCCS) Policlinico San Matteo, Pavia, Italy; ^12^ Department of Oncology, European Institute of Oncology (IEO) Istituto di Ricerca e Cura a Carattere Scientifico (IRCCS), Milan, Italy; ^13^ Immunohematology and Transfusion Service, Fondazione Istituto di Ricerca e Cura a Carattere Scientifico (IRCCS) Policlinico San Matteo, Pavia, Italy; ^14^ Microbiology and Molecular Virology Unit, Fondazione Istituto di Ricerca e Cura a Carattere Scientifico (IRCCS) Policlinico S. Matteo, Pavia, Italy; ^15^ Department of Oncology and Hemato-Oncology, University of Milan, Milan, Italy

**Keywords:** T-cell therapy, nasopharyngeal carcinoma, chemotherapy, epstein-barr virus, autologous cell transplant

## Abstract

**Background:**

Refractory or metastatic nasopharyngeal carcinoma (NPC) patients have a poor prognosis due to the lack of effective salvage treatments and prolonged survival by means of combination chemotherapy being described only for a minority of younger patients with oligometastatic disease. Targeting the Epstein - Barr virus (EBV) proteins expressed in NPC cells has been shown to be a feasible strategy that could help control systemic disease.

**Patients and Methods:**

Between 2011 and 2014, 16 patients with recurrent/metastatic EBV-NPC received first-line chemotherapy (CT) followed by 2 doses of autologous cytotoxic EBV specific T-lymphocytes (15-25 x 10^7^ total cells/dose, 2 weeks apart), based on our previous studies showing the feasibility and efficacy of this infusion regimen. Cumulative overall survival (OS) and median OS were analysed in the whole population and according to specific clinical and biological parameters.

**Results:**

All patients received the planned T-cell therapy schedule, 9 after reaching partial (n=5) or complete (n=4) disease remission with CT, and 7 after failing to obtain benefit from chemotherapy. No severe adverse events were recorded. Patients who received cytotoxic T-lymphocytes (CTLs) had a cumulative 10-year OS of 44%, with a median OS of 60 months (95% CI 42-62). Patients responding to CT, with oligometastatic disease (<3 disease sites), and plasma EBV-DNA <1000 copies/mL had a better outcome.

**Conclusions:**

Autologous EBV-specific CTLs transplanted following conventional first-line CT demonstrated promising efficacy with several patients obtaining long-lasting disease control. The rationale provided by this study, with the crucial role likely played by the timing of CTL administration when trying to induce synergy with conventional treatment needs to be confirmed in a prospective controlled trial.

## Introduction

Nasopharyngeal carcinoma (NPC) presentation varies according to geographic area, with age-standardized incidence rates varying from 3.0 to 0.42 per 100.000 in Asia and Europe, respectively (1). NPC is a highly chemo-radiosensitive disease (2). However, up to 15% of patients develop local failure, and 15% to 30% distant metastasis. The lack of salvage treatment opportunities in these cases portends a poor prognosis (3, 4). Combination chemotherapy with agents such as platinum, taxanes, fluorouracil and gemcitabine results in a median OS of 10 to 15 months, with overall response rates (ORRs) ranging from 25% to 64% (5, 6). The combination of cisplatin and gemcitabine (5) is the first-line treatment. Immunotherapy with checkpoint inhibitors may represent a potential therapeutic tool for patients with metastatic/recurrent NPC, but currently available data do not support their use in everyday clinical practice, as additional prospective studies are needed to define their role in this patient population (6). No standard second-line therapy exists: active agents provide limited clinical benefit (4). Prolonged survival has been described only for a minority of younger patients with oligometastatic disease, the prognosis being related to the number of involved sites and metastases (3, 7).

The involvement of EBV in human carcinogenicity is well-defined (8). Although most EBV-associated tumors are of lymphocyte derivation due to EBV biology, selected epithelial cancers may harbour EBV genome; indeed, in most NPCs, cancer cells express EBV latency antigens (9). Differently from EBV-related post-transplant lymphoproliferative disease (PTLD), NPCs express a restricted set of viral antigens, such as latent membrane protein (LMP) 1 and 2, and EBV nuclear antigens 1 (EBNA1) (9). These immunogens, albeit weak, are capable of inducing a T-lymphocyte response (10, 11), thus NPC cells are recognised by CTLs (12). Based on the success of T-cell therapy for EBV-related PTLD (13), the use of EBV-targeted CTLs (EBV-CTLs) has been receiving attention as a strategy to improve the prognosis of NPC patients (14, 15).

Previous studies have shown the efficacy of EBV-CTLs in refractory NPC patients (16–21). The type and duration of response to cellular immunotherapy may be hampered by the disease burden, which reduces the chances of long-term control.

In the present study, we report on the results of EBV-CTL therapy when administered in an earlier phase of the disease, namely, after receiving first-line CT for recurrent or metastatic NPC.

## Patients and methods

### Patients

Eligible patients were individuals <70 years of age with histologically confirmed, EBV-positive NPC, and an ECOG performance status of 0 or 1, adequate organ function, and measurable lesions according to RECIST (Response Evaluation Criteria in Solid Tumors), version 1.1. Patients undergoing immunosuppressive therapy or with active brain metastases were excluded.

Patients could receive EBV-targeted T-cell therapy as a further treatment after first-line therapy for recurrent or metastatic disease. EBV-CTL treatment was allowed any time after chemotherapy, after having obtained EC authorization (n.15-97). The treatment was conducted in accordance with the International Conference on Harmonization Guidelines on Good Clinical Practice and the Declaration of Helsinki. All patients provided written informed consent before enrolment.

### Production and characterization of EBV-specific CTLs

Peripheral blood mononuclear cells (PBMC) and autologous plasma were collected from all patients through a single leukapheresis, performed before the first-line therapy. EBV-CTLs were expanded *in vitro* following good manufacturing practice (GMP) procedures and cryopreserved following a previously described method (18, 19).

T-cell lines were characterized by immunophenotype, and tested for sterility, alloreactivity, and potency by EBV-specific cytotoxicity by standard ^51^Cr-release assay against a panel of targets, including an autologous B-lymphoblastoid cell line (LCL), and autologous phytohemagglutinin (PHA) blasts pulsed with 2 μg/ml of EBV protein-derived peptide pools (Miltenyi, Bergisch Gladbach, Germany) (18, 19).

### Treatment schedule and patient evaluation

The patients received two doses of autologous EBV-CTLs (15-25 x 10^7^ total cells/dose, 2 weeks apart). After the first infusion, all patients also received low doses of recombinant interleukin-2 (1x10^6^ U subcutaneously daily for three weeks) in order to prolong *in vivo* T-lymphocyte life span.

Patients were monitored for evidence of toxicity by physical examination, serum chemistries and complete and differential blood counts on a weekly basis. Adverse events (AEs) were graded according to the National Cancer Institute Common Terminology Criteria for Adverse Events (NCI CTCAE) Version 4.0. Tumor response was assessed using standard radiographic studies and physical examination according to Response Evaluation Criteria in Solid Tumors (RECIST), version 1.1., and EBV DNA levels (17) on plasma samples at baseline, after each CTL infusion, and every two months thereafter.

### Statistical analysis

Analyses of AEs were descriptive. Clinical benefit was the percentage of patients with complete response (CR), partial response (PR) or prolonged disease stabilization. OS and median OS were calculated using the Kaplan–Meier approach. The difference in survival was analysed by the Log-rank test. All statistical analyses were carried out with Stata 13 (Stata Corporation, College Station, TX, USA) or NCSS System (NCSS, Cary, NC).

## Results

### Patients and EBV-specific CTL line characterization

Between 2011 and 2014, 16 patients with histologically confirmed, EBV-positive undifferentiated NPC received the treatment according to the schedule described. Relevant patient characteristics are reported in **Table 1**.

**Table 1:** Main characteristics and outcomes of the 16 patients enrolled in the study.

Characteristics of patients	Nr.	%
Age at recurrent/metastatic disease, years		
Median (range)	42.5 (17-60)	
Sex		
Male Female	13 3	81 19
ECOG Performance Status (PS)		
0 1	14 2	87.5 12.5
Sites of relapse		
Bone Liver Lung Nodes Local relapse	3 3 4 5 2	19 19 25 31 12.5
Therapy performed at the first line		
TPF Doublets with Cisplatin Gemcitabine/vinorelbine Other	7 5 1 3	4 31 6 19
Response to treatment before CTLs		
PR CR SD PD	5 4 3 4	31 25 19 25
Disease status at last follow up		
NED ED	7 9	44 56

PR, partial response; CR, complete response; SD, stable disease; PD, progressive disease; NED, no evidence of disease; ED, evidence of disease; TPF, cisplatin, docetaxel and 5-fluorouracil.

EBV-CTLs were successfully produced from all patients. Growth kinetics of the T-cell lines from this cohort of NPC patients with disease progression was grossly comparable to those previously observed (19), although in two cases growth was slower and final cellularity lower than in the other cases. Phenotypic analysis indicated, as expected, that the CTL lines included a median of 80% CD3+ T cells (range 40-93), with 20% CD56+/CD3- NK cells (range 6-56). The majority of CD3+ T cells were CD8+ cells (median 69%, range 34-84), but the products contained also a subset of CD4+ T cells (median 18%, range 12-50). EBV-CTLs showed a median EBV-specific cytotoxicity of 46% at an E:T ratio of 10:1 (range 28-77), with median lysis directed to EBV-LMP2 of 13% (range 1-43).

### Patient outcome

No severe AEs were observed in patients receiving the two doses of autologous EBV-CTLs. Only two patients reported mild flu-like syndrome (G1). Nine patients received cell therapy after obtaining partial (PR, n=5) or complete (CR, n=4) disease remission following CT. Eight of these were treated for an oligometastatic disease (less than 3 lesions); one patient with 6 metastatic lesions (bones/nodes) had PR at CT and achieved disease stabilization for 2 years after T-cell therapy. Upon disease progression, she was treated with capecitabine-based CT and reached long-lasting CR. She is alive without evidence of disease at 91 months from diagnosis of metastatic disease. Of the remaining 4 patients treated with EBV-CTLs while in PR after first-line CT, one reached CR after CTL therapy persisting at 5 years (EBV-DNA at the end of CT vs post CTLs: 2430 vs 0 copies/ml), while the others had disease progression after initial stabilization of PR. Two of these patients received radiotherapy and CT followed by cell therapy, respectively, and are in CR at 6- and 5-year follow-ups. Of the 4 patients who reached CR after first-line CT and received adjuvant CTL treatment, 3 persist in CR at 7- 6- and 5-year follow-up, while one patient had disease relapse at 3 months, and reached CR after treatment with 2^nd^-line gemcitabine-based chemotherapy, remaining disease-free for 3 years until new progression. Four patients with progressive disease (PD) and 3 with stable disease (SD), all with more than 2 organs involved and more than 9 metastatic lesions, did not respond to CTL administration and died at a median 12-month follow-up (range 10-48).

As plasma EBV-DNA is a marker of disease in NPC patients, we evaluated patient outcomes also on the basis of this parameter. Before CTL administration, the median plasma EBV-DNA value was 2160 copies/mL (range <10-284384). The baseline value of EBV DNA <1000 copies/mL defined a subgroup of patients with a significantly more favourable outcome (OS: 83% vs 29% in patients with EBV DNA >1000 copies/mL, p<0.01).

**Table 2** summarizes the outcome of patients according to response to CT, disease burden and circulating EBV-DNA values.

**Table 2:** Differences in OS and median OS according to response to chemotherapy, disease burden, and plasma EBV DNA values.

	Median OS months	OS % (95%CI)	p
Response to CT			
CR/PR SD/PD	NR 30	78 (21-100) 0	0.09
Disease burden			
Oligometastatic Non-oligometastatic	NR 47	60 (30-90) 17 (0-46)	<0.05
EBV DNA			
<1000 copies/ml ≥1000 copie/ml	NR 60	83 (54-100) 29 (0-62)	<0.001

CT, chemotherapy; OS, overall survival; CI, confidence interval; CR, complete response; PR, partial response; SD, stable disease; PD, progressive disease; NR, not reached.

The difference in survival was analyzed by the Log-rank test.

## Discussion

We previously demonstrated the feasibility of generating *in vitro* autologous EBV-CTLs from NPC patients that showed *in vitro* antitumor activity and were able to induce disease control once administered *in vivo*. Repeated infusions of up to 1x10^6^ CTLs/kg body weight provided a clinical benefit in heavily pretreated patients (18). To improve the efficacy of our adoptive EBV-specific cell therapy, we subsequently chose to modify our approach by employing both a lymphodepleting regimen and higher doses of EBV-specific autologous CTLs in the same patient population (19). However, the results obtained were substantially comparable with our early experience, with clinical benefit observed in half of the treated patients. Our previous studies (17–19), along with evidence from the literature (20–22), suggest that these cell products could perhaps exert a more robust antitumor effect if employed in earlier phases of the disease i.e as a consolidation after response obtained in patients treated with CT for advanced disease.

**Figure 1:**
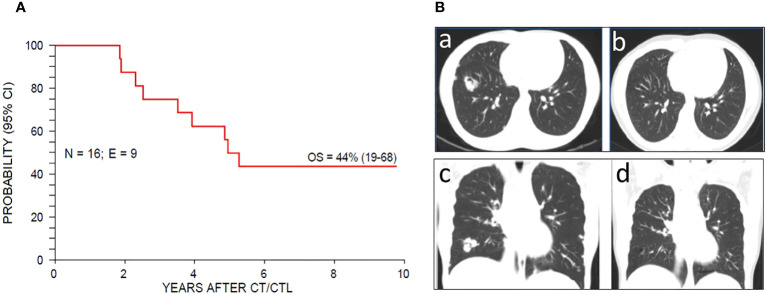
**(A)** Ten-year cumulative incidence OS for the whole population. **(B)** computed tomography scan showing radiological complete remission in a patient with lung metastases. Axial and coronal imaging before CTLs therapy (a, c – baseline) and after 70 months (b, d).

In this paper, we report the results of a retrospective study investigating the efficacy of EBV-CTLs following first-line CT for recurrent/metastatic NPC. The cohort had a cumulative 10-year and median OS that appear significantly better than what was reported for patients treated with systemic therapies without cell therapy (4, 23), and the 29.9 median OS of an endemic NPC cohort treated with chemotherapy and EBV-CTL (21) (**Figure 1**).

Among long-term survivors, three patients who eventually failed EBV-targeted cell therapy reached a CR following conventional treatments and, in one case, subsequent T-cell therapy. This occurrence, supported by previous reports (20, 22), may be explained by a conditioning action of EBV-CTL on tumor cells. Indeed, it has been reported that sublethal death receptor signalling, such as that elicited by CTLs, can enhance tumor cell sensitivity to anticancer drugs (24). In addition, chemotherapy and radiotherapy may expose EBV antigens on tumor cells and induce an immunogenic form of tumor cell death that enhances anticancer immune responses mediated by the transferred CTLs (25). In this regard, the different outcomes between our cohort and that reported by Chia et al. (21) could result from the different timing/schedules between first-line chemo-CTL therapy and subsequent treatments for disease progression.

In line with observations in patients treated at disease onset, in which detectable or high levels of post-radiotherapy plasma EBV-DNA can predict a poor progression-free survival or OS and represents a biomarker of subclinical residual disease (24), patients within our small cohort with plasma EBV-DNA levels ≥1000 cp/ml at the end of first-line CT showed worse outcome. This high-risk group could likely benefit from the association of CT, EBV-CTL therapy, and other biological therapies such as immune checkpoint inhibitors (26–30).

To our knowledge, our paper is the first to report the feasibility and safety of EBV-targeted cell therapy administered following first-line CT for recurrent/metastatic NPC. Moreover, other than confirming that EBV-CTLs possessed *in vitro* antitumor activity and can induce disease control in advanced NPC once administered *in vivo*, our study strengthens our hypothesis that these cell products exert an optimal antitumor effect if employed in earlier phases of the advanced NPC, i.e. as consolidation treatment following response to conventional CT.

The rationale provided by these data, and the importance of the timing of CTL administration following CT, together with possible associations with other biologicals, need to be verified in prospective studies which should include the administration of enriched CTLs specific for the subdominant antigen EBV-LMP2 and other antigens potentially present on tumor cells.

## Data availability statement

The original contributions presented in the study are included in the article/supplementary material. Further inquiries can be directed to the corresponding author.

## Ethics statement

The studies involving human participants were reviewed and approved by Comitato Etico Fondazione IRCCS Policlinico San Matteo, Pavia I. The patients/participants provided their written informed consent to participate in this study. Written informed consent was not obtained from the individual(s) for the publication of any potentially identifiable images or data included in this article.

## Author contributions

Conception and design: PP, SS, SB, PC. Provision of study materials or patients: All Authors Collection and assembly of data: SS, SB, FB, MZ, PC. Data analysis and interpretation: SS, PP, SB, PB, MZ, PC Manuscript writing and final approval: All authors. All authors contributed to the article and approved the submitted version.
